# Association between co-infection with *Chlamydia trachomatis* or *Mycoplasma genitalium* and cervical lesions in HPV-positive population in Hunan, China: a cross-sectional study

**DOI:** 10.1186/s13027-023-00544-5

**Published:** 2023-11-29

**Authors:** Mengjie Jiang, Hui Ding, Ling He, Danning Xu, Ping Jiang, Haoneng Tang, Qian Wang, Xuemei Wang, Lingli Tang

**Affiliations:** 1 Department of Laboratory Medicine, The Second Xiangya Hospital, Central South University, Changsha, Hunan China; 2 Department of Obstetrics and Gynecology, The Second Xiangya Hospital, Central South University, Changsha, Hunan China

**Keywords:** *Chlamydia trachomatis*, *Mycoplasma genitalium*, HPV, STIs, Cervical lesions, China

## Abstract

**Objectives:**

The aim of this study was to determine the prevalence of *Chlamydia trachomatis* (CT) and *Mycoplasma genitalium* (MG) among HPV-positive women undergoing colposcopy at the Second Xiangya Hospital of Central South University, Hunan, China. Additionally, we aimed to assess the impact of *C. trachomatis* or *M. genitalium* co-infection with HPV on the severity of cervical lesions.

**Methods:**

We collected HPV data, cervical cytology results, and demographic information from 439 women attending colposcopy. Cervical swabs were obtained for simultaneous amplification testing (SAT) of *C. trachomatis* and *M. genitalium*. Multivariate logistic regression analyses were performed to examine the association between sexually transmitted pathogens and cervical lesions.

**Results:**

Among the participants, *C. trachomatis* was detected in 17 (3.87%) individuals, and *M. genitalium* in 16 (3.64%) individuals. There was no co-infection of *C. trachomatis* and *M. genitalium*. The highest prevalence of *M. genitalium* was observed in women aged 19–30 years (10.20%; 95% CI, 1.41-18.99%), with a subsequent decline in prevalence with increasing age (Ptrend = 0.014). The most common HPV subtype in our study was HPV52 (30.79%), followed by HPV16 (18.62%), HPV58 (16.95%), and HPV53 (10.02%). Infection with HPV16 (OR = 3.43, 95% CI, 2.13–5.53), HPV31 (OR = 3.70, 95% CI, 1.44–9.50), and HPV33 (OR = 3.71, 95% CI, 1.43–9.67) was associated with an increased severity of cervical lesions, while HPV53 infection was not likely to lead to advanced cervical lesions (OR = 0.45, 95% CI, 0.23–0.89). The leukocyte level in vaginal secretions (P = 0.042) and cervical cytology results (P < 0.001) showed associations with the degree of cervical lesions. However, there was no significant association between *C. trachomatis* or *M. genitalium* infection and the severity of cervical lesions, nor with their co-infection with HPV16.

**Conclusions:**

There was no correlation between co-infection of *Chlamydia trachomatis* or *Mycoplasma genitalium* and the degree of cervical lesions in HPV-positive population in Hunan, China. Our findings emphasized the need to pay more attention to *M. genitalium* infection among young women. Increased levels of leukocytes in vaginal secretions may be linked to cervical lesions. HPV16, HPV31, and HPV33 in Hunan province, China, may exhibit higher cervical pathogenicity.

## Introduction

In early 2021, the GLOBOCAN 2020 database, produced by the International Agency for Research on Cancer (IARC), revealed that cervical cancer (CC) ranks as the fourth most common cancer worldwide among women in terms of both incidence and mortality [1]. In China, it stands as the ninth most common cancer type [2]. Persistent infection with high-risk human papillomavirus (HPV) is a key factor contributing to the development and progression of cervical cancer [3]. While the majority of women contract HPV at some point in their lives, most cases resolve spontaneously, and only a small percentage of persistent infections lead to cervical lesions or cancer [4]. Therefore, it is important to focus on the risk factors that contribute to the progression of HPV infection to cervical cancer. The prevalence and pathogenicity of different HPV subtypes that lead to precancerous lesions and cervical cancer can vary among countries and populations [5]. Hence, it is crucial to determine the prevalence and impact of each HPV type in a specific region to effectively control and eliminate HPV infections, as well as prevent cervical lesions.

Sexually transmitted pathogens pose a significant public health concern worldwide, particularly for women’s health. *Chlamydia trachomatis* (CT) is the most common causative agent of bacterial sexually transmitted infections (STIs) and can result in various complications in women, including pelvic inflammatory disease (PID), hydrosalpinx, and infertility [6]. Some studies have suggested an association between *C. trachomatis* infection and HPV infection, indicating they are mutual risk factors [7, 8]. However, conflicting conclusions have also been reported. A cross-sectional study conducted in Beijing, China, found no significant difference in *C. trachomatis* infection rates between HPV-infected and non-HPV-infected groups or between groups with different cervical biopsy results [9]. Similarly, a retrospective analysis conducted in Sichuan, China, found no association between *C. trachomatis* infection and high-risk HPV infection [10]. Therefore, the relationship between *C. trachomatis* and HPV infection remains controversial and requires further investigation. Additionally, most existing studies focus solely on the relationship between *C. trachomatis* infection and HPV infection, disregarding the fact that HPV infection does not necessarily lead to cervical lesions [3]. Therefore, exploring the relationship between *C. trachomatis* infection and cervical lesions directly reflects the impact of *C. trachomatis* on female cervical health, yet few relevant studies have been conducted.

*Mycoplasma genitalium* (MG) is an emerging sexually transmitted pathogen that causes genitourinary tract diseases and has recently gained attention [11]. Research has indicated that *M. genitalium* can cause cervicitis [6] and is associated with various gynecological conditions, including PID [12] and infertility [13]. Furthermore, most *M. genitalium* infections are asymptomatic. The increasing prevalence and antimicrobial resistance of *M. genitalium* are concerning [6]. However, routine testing for *M. genitalium* is not commonly conducted in most hospitals in China, and epidemiological data on *M. genitalium* in Hunan Province is lacking. The relationship between *M. genitalium*, HPV progression, and cervical lesions remains unknown but is of significant importance.

Currently, there is inconsistency in the association between *C. trachomatis* infection and HPV infection. Moreover, epidemiological data on *M. genitalium* and its correlation with HPV infection are limited. Therefore, we conducted a study among HPV-positive individuals who underwent colposcopy in Hunan Province, China. This study aimed to investigate the distribution of HPV subtypes, as well as the prevalence and age distribution of *C. trachomatis* and *M. genitalium*. Additionally, it analyzed the risk factors for cervical lesions and examined the influence of co-infection with *C. trachomatis* or *M. genitalium* on cervical lesions in HPV-infected individuals. These findings have important implications for the prevention and control of infections caused by these pathogens, as well as the management of cervical lesions.

## Materials and methods

### Study design

A cross-sectional study was carried out at the Second Xiangya Hospital of Central South University from July to September 2022. A population of 500 women visiting the hospital’s gynecology department and undergoing colposcopy was invited to participate in the study. The inclusion criteria were as follows: (1) women with a history of sexual activity, (2) positive HPV test results, and (3) informed consent. The exclusion criteria included: (1) women infected with bacterial vaginosis (BV), fungi, or *Trichomonas vaginalis* (TV) at the time of examination, (2) women had sexual activity or gynecological examinations within the past 24 h, (3) women who are menstruating, (4) pregnant women, (5) women who had vaginal douching or used vaginal medications within the past 48 h, (6) women who had undergone hysterectomy, (7) women who had undergone cervical loop electrosurgical excision procedure (LEEP), laser treatment, or cold knife conization (CKC) within the past year, and (8) women with incomplete laboratory measurements. Following the application of these criteria, a total of 439 women (aged 19 to 74 years) were included in the final analysis. The study protocol was approved by the institutional review board of the Second Xiangya Hospital of Central South University (approval number: LYF2022113) and by the Chinese medical research registration information system (Ref number: MR-43-23-027426). Written informed consent was ensured from all study participants to take part in the study voluntarily after they get informed about the objective and purpose of the study.

### Study procedure and sample collection

The study participants underwent colposcopy performed by a single trained gynecologist. After inserting a vaginal dilator to fully expose the cervix, a flocked swab (SanEn biotechnology Co., Ltd, Tianjin, China) was inserted into the cervix and rotated several times to collect cervical secretions for the detection of *C. trachomatis* and *M. genitalium*. The cervical swab was then placed into 2 ml of a transport and preservation medium (Rendu Biotechnology, Shanghai, China) and immediately frozen at -80℃. Acetic acid and Lugol iodine were applied to examine the cervix and vaginal walls. Any suspicious areas were biopsied for histopathological assessment. The histopathological grading included no squamous intraepithelial lesions (NO SIL), low-grade squamous intraepithelial lesions (LSIL), high-grade squamous intraepithelial lesions (HSIL), and cervical cancer. If multiple biopsy samples were taken simultaneously, the sample with the highest grade was considered for analysis. The researchers will track and record the biopsy results of each patient in the hospital’s medical record system using the patient’s unique card number.

Before undergoing colposcopy, the patient’s laboratory measurements, including HPV types, cervical cytology tests, and the level of vaginal leukocytes, as well as demographic information such as age, pregnancy history, reproductive history, and menopause, were recorded.

### Laboratory measurements

Laboratory examination data were collected when the subjects underwent colposcopy.

HPV testing was performed using the Human Papillomavirus nucleic acid typing test kit (Toujing, Shanghai, China), which allowed for the detection of 21 HPV types. Among these, 15 HPV types were classified as high-risk HPV (HR-HPV: 16, 18, 31, 33, 35, 39, 45, 51, 52, 53, 56, 58, 59, 66, and 68), and 6 HPV types were classified as low-risk HPV (LR-HPV: 6, 11, 42, 43, 44, and 81).

The pathologists categorized the results of the cervical cytology as follows: Negative for intraepithelial lesion or malignancy (NILM), Atypical squamous cells of undetermined significance (ASC-US), Atypical squamous cells: cannot exclude high-grade squamous intraepithelial lesion (ASC-H), Low-grade squamous intraepithelial lesion (LSIL), High-grade squamous intraepithelial lesion (HSIL), Atypical glandular cells (AGC) and Squamous cell carcinoma (SCC). None of the patients participating in this study had been diagnosed with AGC. Therefore, the AGC cytological grade is not listed in Table 1.

The levels of leukocytes in the vaginal secretions of the participants were examined by gynecologists. Based on the number of leukocytes observed in each high-power field, the examination results were classified into three categories: 0–15/HP, 16–30/HP, and > 30/HP.

All laboratory measurements were obtained from the Second Xiangya Hospital, Central South University.

### **Simultaneous Amplification and Testing (SAT) assay for*C. trachomatis*and*M. genitalium*detection**

In a biosafety cabinet, the swab soaked in the preservation solution was fully ground and then discarded. The same volume of normal saline was added and thoroughly mixed. The target RNA was extracted using magnetic beads by adding nucleic acid extraction solution to the appropriate samples, following the instructions provided by the *C. trachomatis* and *M. genitalium* commercial test kits (Rendu Biotechnology, Shanghai, China). The magnetic beads containing the target nucleic acid were mixed with the prepared amplification detection liquid. A 30µL magnetic bead suspension was placed into each microreaction tube, and 10µL of preheated enzyme solution was added to start the reaction. The reaction process was set at 42℃ for 1 min per cycle, with a total of 40 cycles. The fluorescence signals in the fluorescein FAM channel were recorded every minute. A Ct value of ≤ 35 indicated a positive result for *C. trachomatis* RNA or *M. genitalium* RNA. For samples with Ct values between 35 and 40, retesting was required. A Ct value of < 40 in the retesting was considered positive.

### Statistical analyses

All statistical analyses were conducted using SPSS version 25.0 (Chicago, IL, United States). The Shapiro-Wilk test was employed to assess the normal distribution of variables. Continuous variables that were not normally distributed are presented as the Median (interquartile range, IQR: Q1-Q3), while categorical variables are presented as percentages. The Chi-square test or nonparametric Kruskal-Wallis test was used to compare the three groups statistically. Chi-square trend tests were used to examine changes in the prevalence of *C. trachomatis* and *M. genitalium* by age. A multivariate logistic regression model was employed to evaluate factors associated with cervical lesions using variables with a P-value of less than 0.1 in univariate analyses. Throughout the study, results were considered statistically significant when P ≤ 0.05.

**Fig. 1:**
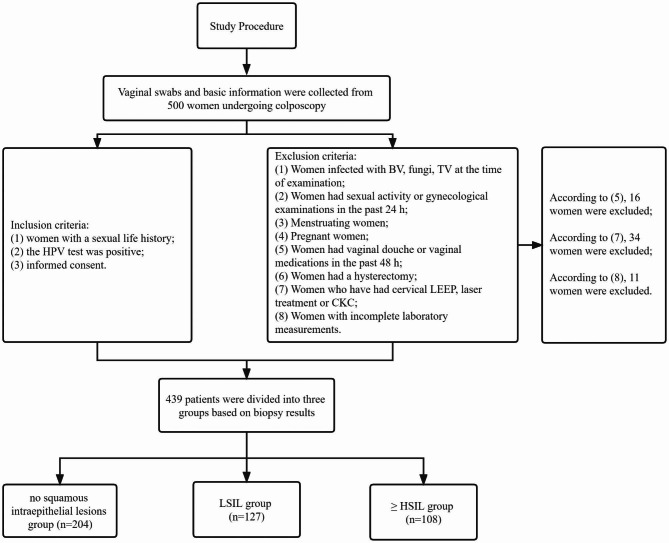
Flow chart of the study procedure

## Results

### Socio-demographic characteristics among participants

According to the inclusion and exclusion criteria, a total of 439 out of the 500 individuals who underwent colposcopy were included in our study **(**Fig. 1**)**. The median age of the participants was 45 years (IQR 36–53). Based on cervical biopsy results, the 439 HPV-positive patients were categorized into three groups: no squamous intraepithelial lesions group (NO SIL, n = 204), low-grade squamous intraepithelial lesion (LSIL) group (n = 127), and high-grade squamous intraepithelial lesion (≥ HSIL) group (n = 108). Table 1 presents the baseline characteristics of the participants. Among the participants, *C. trachomatis* was detected in 17 individuals (3.87%), *M. genitalium* in 16 individuals (3.64%), and there was no co-infection of *C. trachomatis* and *M. genitalium*. Significant differences were observed in the leukocyte level of vaginal secretions among the groups (P = 0.042), with the highest leukocyte level (> 30/HP) observed in the HSIL group (12.38%). Cervical cytology results also showed significant differences among the groups (P < 0.001), with the NILM grade being more common in the group without complicated lesions (81.86%), while the ASC-H (10.19%) and HSIL (15.74%) grades were more common in the HSIL group. No significant differences were found in age, pregnancy history, reproductive history, menopause, HPV single or mixed infection, or the positive rate of *C. trachomatis* or *M. genitalium* among the groups.

**Table 1:** Baseline characteristics of 439 women undergoing colposcopy in the Second Xiangya Hospital of Central South University according to the degree of cervical lesions

Variables	All (n = 439)		NO SIL (n = 204)		LSIL (n = 127)		≥HSIL (n = 108)		P value
Age, years, median (IQR)	439	45.00 (36.00–53.00)	204	46.00 (37.25-53.00)	127	45.00 (34.00–53.00)	108	41.00 (35.00-52.75)	0.437
19–30, n (%)		49 (11.16)		20 (9.80)		20 (9.80)		14 (12.96)	0.421
31–40, n (%)		116 (26.42)		50 (24.51)		50 (24.51)		34 (31.48)	
41–50, n (%)		119 (27.11)		59 (28.92)		59 (28.92)		27 (25.00)	
51–60, n (%)		130 (29.61)		68 (33.33)		68 (33.33)		24 (22.22)	
> 60, n (%)		25 (5.69)		7 (3.43)		7 (3.43)		9 (8.33)	
History of pregnancy, n (%)	439		204		127		108		
Yes		409 (93.17)		193 (94.61)		114 (89.76)		102 (94.44)	0.197
No		30 (6.83)		11 (5.39)		13 (10.24)		6 (5.56)	
Number of pregnancy, n (%)	423		197		121		105		
0		30 (7.09)		11 (5.58)		13 (10.72)		6 (5.71)	0.376
1		50 (11.82)		24 (12.18)		15 (12.40)		11 (10.48)	
2		93 (21.99)		41 (20.81)		24 (19.83)		28 (26.67)	
3		85 (20.09)		38 (19.29)		27 (22.31)		20 (19.05)	
4		75 (17.73)		36 (18.27)		18 (14.88)		21 (20.00)	
≥ 5		90 (21.28)		47 (23.86)		24 (19.83)		19 (18.10)	
History of production, n (%)	439		204		127		108		
Yes		385 (87.70)		183 (89.71)		106 (83.46)		96 (88.89)	0.222
No		54 (12.30)		21 (10.29)		21 (16.54)		12 (11.11)	
Parity, n (%)	424		197		122		105		
0		54 (12.74)		21 (10.66)		21 (17.21)		12 (11.43)	0.435
1		173 (40.80)		93 (47.21)		41 (33.61)		39 (37.14)	
2		156 (36.79)		65 (32.99)		50 (40.98)		41 (39.05)	
≥ 3		41 (9.67)		18 (9.14)		10 (8.20)		13 (12.38)	
Menopause, n (%)	439		204		127		108		
Yes		164 (37.36)		79 (38.73)		47 (37.01)		38 (35.19)	0.824
No		275 (62.64)		125 (61.27)		80 (62.99)		70 (64.81)	
Leukocyte level, n (%)	432		203		124		105		
0–15/HP		217 (50.23)		114 (56.16)		53 (42.74)		50 (47.62)	0.042
16–30/HP		175 (40.51)		75 (36.94)		58 (46.78)		42 (40.00)	
> 30/HP		40 (9.26)		14 (6.90)		13 (10.48)		13 (12.38)	
HPV infection types, n (%)	419		196		124		99		
Single infection		314 (74.94)		146 (74.49)		87 (70.16)		81 (81.82)	0.134
Multiple infection		105 (25.06)		50 (25.51)		37 (29.84)		18 (18.18)	
Cervical cytology test, n (%)	439		204		127		108		< 0.001
NILM		274 (62.41)		167 (81.86)		69 (54.34)		38 (35.18)	
ASC-US		98 (22.32)		26 (12.75)		41 (32.28)		31 (28.70)	
ASC-H		11 (2.51)		0 (0.00)		0 (0.00)		11 (10.19)	
LSIL		37 (8.43)		11 (5,39)		15 (11.81)		11 (10.19)	
HSIL		19 (4.33)		0 (0.00)		2 (1.57)		17 (15.74)	
CT, n (%)	439	17 (3.87)	204	10 (4.90)	127	3 (2.36)	108	4 (3.70)	0.505
MG, n (%)	439	16 (3.64)	204	10 (4.90)	127	3 (2.36)	108	3 (2.78)	0.418

Data are presented as the Median (IQR: Q1–Q3) for continuous variables that are not normally distributed and percentage for categorical variables. The Chi-square test or nonparametric Kruskal-Wallis test were used for statistical comparisons between three groups. P value ≤ 0.05 was considered statistically significant

NILM: Negative for intraepithelial lesion or malignancy; ASC-US: Atypical squamous cells of undetermined significance; ASC-H: Atypical squamous cells: cannot exclude high-grade squamous intraepithelial lesion; LSIL: Low-grade squamous intraepithelial lesion; HSIL: High-grade squamous intraepithelial lesion; IQR: interquartile range; CT: *Chlamydia trachomatis*; MG: *Mycoplasma genitalium*

### **The prevalence of *M. genitalium* decreased with increasing age, but the prevalence of *C. trachomatis* did not**

Subjects in our study were stratified by age (ranging from 19 to 74 years), and we examined the changes in the prevalence of *C. trachomatis* and *M. genitalium* with respect to age. The overall prevalence of *M. genitalium* was 3.64% (95% CI, 1.88-5.40%), with statistically significant differences observed among different age groups. The highest prevalence was found in women aged 19–30 years (10.20%; 95% CI, 1.41-18.99%), and it steadily decreased as women aged, reaching 1.94% (95% CI, 0.26-4.13%) in women over 50 years old (Ptrend = 0.014; Table 2). Surprisingly, the prevalence of *C. trachomatis* was higher in women aged 41–50 years (4.20%; 95% CI, 0.54-7.86%) and in women over 50 years old (5.16%; 95% CI, 1.64-8.68%), and it did not show a trend with increasing age (Ptrend = 0.301).

**Table 2:** The prevalence of *Mycoplasma genitalium* and *Chlamydia trachomatis* changes with age

Age (years)	n	*M. genitalium* Prevalence, % (95% CI)	*C. trachomatis* Prevalence, % (95% CI)
All	439	3.64 (1.88–5.40)	3.87 (2.06–5.68)
19–30	49	10.20 (1.41–18.99)	4.08 (1.67–9.82)
31–40	116	4.31 (0.56–8.06)	1.72 (0.68–4.13)
41–50	119	2.52 (0.34–5.38)	4.20 (0.54–7.86)
> 50	155	1.94 (0.26–4.13)	5.16 (1.64–8.68)
Ptrend		0.014	0.301

Ptrend was obtained by Chi-square trend tests, Ptrend ≤ 0.05 was considered statistically significant

CI: confidence interval

### **The prevalence of *C. trachomatis* or *M. genitalium* was not associated with the degree of cervical lesions**

Multivariate logistic regression analyses were conducted to investigate the association between *C. trachomatis* or *M. genitalium* and the severity of cervical lesions **(**Table 3**)**. The results showed that neither LSIL nor HSIL phenotypes were associated with either *C. trachomatis* or *M. genitalium* in any of the models, using the NO SIL phenotype as the reference. After adjusting for all confounding factors, the prevalence of *C. trachomatis* or *M. genitalium* was not found to be associated with the severity of cervical lesions (all P > 0.05).

**Table 3:** Multivariable logistic regression analysis of the association between *C. trachomatis* or *M. genitalium* and the degree of cervical lesions

Models	NO SIL	LSIL		≥HSIL	
		OR (95% CI)	P value	OR (95% CI)	P value
*C. trachomatis*					
Model 1	1	0.469 (0.127–1.739)	0.258	0.746 (0.228–2.437)	0.628
Model 2	1	0.532 (0.141–2.012)	0.352	0.704 (0.209–2.372)	0.571
Model 3	1	0.581 (0.138–2.443)	0.459	1.156 (0.255–5.240)	0.851
*M. genitalium*					
Model 1	1	0.469 (0.127–1.739)	0.258	0.554 (0.149–2.058)	0.378
Model 2	1	0.364 (0.092–1.444)	0.151	0.510 (0.131–1.987)	0.332
Model 3	1	0.414 (0.086–1.991)	0.271	1.110 (0.177–6.974)	0.911

Model 1 was unadjusted; Model 2 was adjusted for age, pregnancy history, number of pregnancy, reproductive history, parity and menopause; Model 3 was adjusted for model 2 plus leukocyte level, HPV infection types, cervical cytology test and *M. genitalium* or *C. trachomatis*

OR: Odds Ratio; CI: confidence interval

### Association of human papillomavirus with the degree of cervical lesions

Out of the 439 women included in our study, 20 had untyped HPV test results, so we analyzed accurate HPV typing results for 419 women. The most prevalent HPV genotype was HPV52 in 129 subjects (30.79%), followed by HPV16 in 78 subjects (18.62%), HPV58 in 71 subjects (16.95%), and HPV53 in 42 subjects (10.02%). In the univariate analysis, HPV genotypes associated with cervical lesions were HPV16 (OR 3.35, 95% CI 2.10–5.33; P = 0.000), HPV31 (OR 2.67, 95% CI 1.05–6.76; P = 0.039), and HPV33 (OR 3.17, 95% CI 1.24–8.13; P = 0.016), indicating that they increased the severity of cervical lesions. Surprisingly, patients infected with HPV53 were less likely to develop advanced cervical lesions (OR 0.39, 95% CI 0.20–0.76; P = 0.006; Table 4). Due to the low number of detected cases of HPV45 and HPV11, which only appeared in the NO SIL group but not in the LSIL and HSIL groups, statistical analysis could not be conducted. In the multivariate analysis including HPV genotypes with a P-value < 0.10, the severity of cervical lesions was associated with HPV16, HPV31, HPV33, and HPV53.

**Table 4:** Relationship of human papillomavirus and the degree of cervical lesions by univariate and multivariate logistic regression analysis

HPV Genotype	n (%)	Univariate analysis		Multivariate analysis	
		OR (95% CI)	P value	OR (95% CI)	P value
HPV16	78 (18.62)	3.35 (2.10–5.33)	0.000	3.43 (2.13–5.53)	0.000
HPV18	30 (7.16)	1.03 (0.51–2.05)	0.943	-	-
HPV31	16 (3.82)	2.67 (1.05–6.76)	0.039	3.70 (1.44–9.50)	0.007
HPV33	16 (3.82)	3.17 (1.24–8.13)	0.016	3.71 (1.43–9.67)	0.007
HPV35	4 (0.95)	3.06 (0.48–19.47)	0.236	-	-
HPV39	30 (7.16)	0.71 (0.33–1.39)	0.289	-	-
HPV45	3 (0.72)	-*	-*	-	-
HPV51	32 (7.64)	0.64 (0.32–1.29)	0.214	-	-
HPV52	129 (30.79)	1.01 (0.69–1.49)	0.963	-	-
HPV53	42 (10.02)	0.39 (0.20–0.76)	0.006	0.45 (0.23–0.89)	0.022
HPV56	13 (3.10)	0.49 (0.16–1.49)	0.207	-	-
HPV58	71 (16.95)	0.97 (0.60–1.57)	0.906	-	-
HPV59	9 (2.15)	0.85 (0.25–2.98)	0.805	-	-
HPV66	16 (3.82)	0.68 (0.26–1.78)	0.431	-	-
HPV68	18 (4.30)	0.42 (0.16–1.12)	0.082	0.48 (0.17–1.30)	0.148
HPV6	8 (1.91)	0.63 (0.16–2.46)	0.502	-	-
HPV11	4 (0.95)	-*	-*	-	-
HPV42	12 (2.86)	1.07 (0.37–3.10)	0.908	-	-
HPV43	7 (1.67)	0.37 (0.07–1.84)	0.224	-	-
HPV44	4 (0.95)	0.26 (0.03–2.64)	0.255	-	-
HPV81	23 (5.49)	0.46 (0.20–1.09)	0.079	0.47 (0.20–1.14)	0.095

Factors with P < 0.10 on univariate logistic regression analyses were included in the multiple logistic regression model. –*, parameter couldn’t get P value or odds ratio due to the low number of cases detected; –, parameter not included in multivariate analysis

OR: Odds Ratio; CI: confidence interval

### Association of sexually transmitted infections and human papillomavirus infection with the degree of cervical lesions

Having identified the correlation between HPV16, HPV31, HPV33, and HPV53 with the extent of cervical lesions **(**Table 4**)**, we further explored the combined effects of sexually transmitted infections (STIs) and HPV genotypes on the severity of cervical lesions. Due to the small number of positive cases of *C. trachomatis* and *M. genitalium*, and the fact that none of the patients infected with HPV31 and HPV33 were infected with *C. trachomatis* or *M. genitalium*, we only investigated the relationship between HPV16 or HPV53 and *C. trachomatis* or *M. genitalium* (Table 5). In the univariate logistic regression analysis, we did not observe any significant interactions between *C. trachomatis* or *M. genitalium* and HPV16 (all P > 0.05). Since patients infected with HPV53 rarely had *C. trachomatis* infection, and co-infection was only present in the NO SIL group but not in the LSIL and HSIL groups, statistical analysis could not be performed. Furthermore, no significant interaction was found between HPV53 and *M. genitalium* (P > 0.05).

**Table 5:** Analysis of the joint effect of sexually transmitted infections and human papillomavirus genotypes on the degree of cervical lesions

Variables	n	NO SIL	LSIL	≥ HSIL	OR (95% CI)	P value
HPV16 with CT	7	2	1	4	1.35 (0.31–5.94)	0.689
HPV16 without CT	71	19	20	32		
HPV16 with MG	2	1	0	1	0.65 (0.05–8.64)	0.744
HPV16 without MG	76	20	21	35		
HPV53 with CT	1	1	0	0	-*	-*
HPV53 without CT	41	26	13	2		
HPV53 with MG	2	1	1	0	1.60 (0.10-25.87)	0.741
HPV53 without MG	40	26	12	2		

–*, parameter couldn’t get P value or odds ratio due to the low number of cases detected

CT: *Chlamydia trachomatis*; MG: *Mycoplasma genitalium*; OR: Odds Ratio; CI: confidence interval

## Discussion

A cross-sectional study was conducted among 439 HPV-positive women who underwent colposcopy in Hunan province, China, to investigate the prevalence and age distribution of *C. trachomatis* and *M. genitalium* infections, as well as the impact of co-infection with HPV on cervical lesions. The aim of the study was to contribute to the existing research in this field. *C. trachomatis* was detected in 17 participants (3.87%), while *M. genitalium* was detected in 16 participants (3.64%). Co-infection of *C. trachomatis* and *M. genitalium* was not observed. The prevalence of *M. genitalium* was highest in women aged 19–30 years (10.20%; 95% CI, 1.41-18.99%) and decreased steadily with age (Ptrend = 0.014). It was observed that HPV16, HPV31, and HPV33 may exhibit higher cervical pathogenicity in Hunan province, China. Additionally, a surprising finding was the association between elevated levels of leukocytes in vaginal secretions and cervical lesions. However, neither *C. trachomatis* nor *M. genitalium* infection, alone or in co-infection with HPV16, were found to be associated with the severity of cervical lesions. This study provided insights into the pathogenicity of different HPV subtypes on cervical lesions and explored the age distribution of *M. genitalium* and its relationship with cervical lesions in the HPV-positive population in Hunan province, China. These findings are expected to contribute to the understanding of the epidemic pathology of sexually transmitted pathogens and aid in the prevention, screening, and control of such pathogens.

The population included in our study had certain unique characteristics. Samples were collected from the colposcopy laboratory, and patients who underwent colposcopy were excluded if they had bacterial vaginitis, fungal vaginitis, trichomonas vaginitis, HIV, or syphilis. This exclusion was done to prevent worsening of the patients’ condition and to avoid the risk of infection for healthcare workers. Therefore, bacterial, fungal, trichomonas, HIV, and syphilis infections were not considered in our study population to eliminate any potential synergistic pathogenic effects they might have with *C. trachomatis* or *M. genitalium*.

The prevalence of both *C. trachomatis* and *M. genitalium* was relatively low, with *C. trachomatis* often having a higher prevalence compared to *M. genitalium*. For instance, among the general female population visiting gynecology departments in Sichuan province, China, the infection rates of *C. trachomatis* and *M. genitalium* were reported as 6.5% and 2.6%, respectively [10]. In a study on women undergoing cervical cancer screening in Beijing, China, the infection rates of *C. trachomatis* and *M. genitalium* were 11.3% and 1.0%, respectively [9]. In our study, the infection rate of *C. trachomatis* (3.87%) was slightly lower than that reported in other studies, while the infection rate of *M. genitalium* (3.64%) was slightly higher. Co-infection of *M. genitalium* with other sexually transmitted pathogens has been reported. In young high-risk women with asymptomatic bacterial vaginosis in the United States, the prevalence of *M. genitalium* co-infection with *C. trachomatis* was 29.9%, and co-infection with *Neisseria gonorrhoeae* was 23.6% [14]. However, in a study conducted in Belgium, Germany, Spain, and the United Kingdom, the co-infection rate of *M. genitalium* with *C. trachomatis* was only 0.6%, and with *N. gonorrhoeae* was 0.1% [15]. In our study, co-infection of *C. trachomatis* and *M. genitalium* was not observed, likely due to differences in the study population and the low positive rates of *C. trachomatis* and *M. genitalium*.

Studies have indicated that sexually transmitted pathogen infections are more prevalent among young women [6]. Our study revealed that the highest prevalence of *M. genitalium* was observed in women aged 19 to 30 years (10.20%; 95% CI, 1.41–18.99%), and this prevalence declined steadily with age, consistent with previous research [16, 17]. However, the prevalence of *C. trachomatis* was higher among women over 50 years old (5.16%; 95% CI, 1.64–8.68%), but not in young women under 30 years of age. This finding contradicts Chen’s research, as Chen et al. discovered that the prevalence of *C. trachomatis* infection was highest among the group aged ≤ 25 years and gradually decreased with age [8]. However, a study conducted in Shenzhen, China, found that the prevalence of *C. trachomatis* infection was higher in the group aged > 35 years compared to the group aged ≤ 35 years [18]. This finding aligns with our study and suggests that *C. trachomatis* is not necessarily exclusive to young women. The inconsistent results may be attributed to differences in study areas and populations. In conclusion, our findings suggest that the growing openness towards sexual concepts and the younger age at first sexual intercourse among Chinese women may increase the likelihood of sexually transmitted pathogen infections in young women, but the health of elderly women should not be disregarded.

Few studies have explored the relationship between leukocyte levels in vaginal secretions and the severity of cervical lesions. A study conducted in southwest China found that patients infected with *C. trachomatis* or *M. genitalium* were more likely to have elevated leukocyte levels in vaginal secretions [10]. In our study, we observed that increased leukocyte levels in vaginal secretions may be associated with cervical lesions. Leukocyte levels are often indicative of inflammation, but our study excluded patients with bacterial vaginitis, fungal vaginitis, and trichomonal vaginitis. The elevated leukocyte levels in the vaginal secretions of the subjects may have resulted from an imbalance in the vaginal microecology. In recent years, numerous studies have focused on the relationship between vaginal microbiota and female reproductive tract diseases [19, 20]. Some studies have revealed that an imbalance in vaginal microecology may be associated with persistent HPV infection and cervical lesions [21, 22]. In the future, metagenomics technology can be employed to further investigate the vaginal microbiota and its relationship with cervical lesions.

Persistent infection with high-risk HPV can result in cervical lesions and potentially cervical cancer. The prevalence and distribution of HPV subtypes vary across countries, races, and populations. In our study, the most prevalent HPV genotype was HPV52 (30.79%), followed by HPV16 (18.62%), HPV58 (16.95%), and HPV53 (10.02%). These results align with data from other Chinese populations. A study conducted in southern China identified HPV52, HPV16, and HPV58 as the three most common HPV subtypes [8]. In the Inner Mongolia region of China, HPV16 was the most prevalent genotype, followed by HPV58 and HPV52 [23]. This indicates that HPV16, HPV52, and HPV58 are predominant among the general Chinese population. However, a high prevalence of HPV subtypes does not necessarily imply their strong ability to cause cervical lesions. In our study, HPV16 (OR = 3.43, 95% CI, 2.13–5.53), HPV31 (OR = 3.70, 95% CI, 1.44–9.50), and HPV33 (OR = 3.71, 95% CI, 1.43–9.67) infections were associated with an increased severity of cervical lesions, whereas HPV53 infection was not likely to progress to advanced cervical lesions (OR = 0.45, 95% CI, 0.23–0.89). Therefore, it is recommended to pay more attention to patients infected with more pathogenic HPV subtypes based on local HPV epidemiological data when managing HPV-positive patients.

Many previous studies have primarily focused on the relationship between cervical cytology results and sexually transmitted pathogens [23, 24]. However, cervical cytology results do not fully represent the cervical status. Cervical biopsy serves as the gold standard for assessing cervical status, highlighting the importance of exploring the relationship between sexually transmitted pathogens, HPV infection, and cervical biopsy. In our study, we found no association between *C. trachomatis* and *M. genitalium* infection, either alone or in co-infection with HPV16, and the severity of cervical lesions. Regarding *C. trachomatis* infection, its association with HPV infection and cervical lesions is currently controversial. Several studies have indicated an association between *C. trachomatis* infection and HPV infection as well as cervical lesions [8, 23]. However, other studies have suggested that *C. trachomatis* infection does not increase the risk of HPV infection and cervical lesions [9, 10, 25], which aligns with our research findings. The conflicting results may stem from differences in cohorts or limitations in sample size. As for *M. genitalium* infection, there is limited research on its association with HPV infection and cervical lesions. A study by A et al. found no association between *M. genitalium* infection and HPV infection or cervical lesions [9], which is consistent with our findings. Currently, most studies are cross-sectional or retrospective. Large-scale, multicenter prospective cohort studies are warranted to explore the relationship between co-infection of sexually transmitted pathogens with HPV and cervical lesions.

This study had several limitations. Firstly, it was a cross-sectional study, which cannot accurately capture the dynamic changes in HPV infection and cervical lesions among the subjects. Secondly, the sample size was small, and due to the low infection rates of *C. trachomatis* and *M. genitalium*, the number of positive cases was insufficient to conduct comprehensive stratified analysis. Additionally, the samples collected were primarily from Changsha city, Hunan Province, which may affect the generalizability of the findings regarding the prevalence of sexually transmitted pathogens. Future research should be conducted using large longitudinal cohorts, including women from different locations, to identify and validate risk factors for HPV infection and the progression of cervical lesions, ultimately aiming to prevent cervical cancer.

In conclusion, our study suggests that greater attention should be given to *M. genitalium* infection among young women, while *C. trachomatis* infection in older women should not be overlooked. Increased levels of leukocytes in vaginal secretions may be associated with cervical lesions. In Hunan province of China, HPV16, HPV31, and HPV33 appear to have higher pathogenicity in relation to cervical lesions. The findings of this study highlight the ongoing controversy regarding whether *C. trachomatis* and *M. genitalium* infection, either alone or in co-infection with HPV, contribute to an increased severity of cervical lesions. Further clarification is needed through large longitudinal cohort studies in the future.

## Data Availability

The original data source could be shared upon the request of the principal investigator.
